# The effect of distance to health facility on neonatal mortality in Ethiopia

**DOI:** 10.1186/s12913-023-09070-x

**Published:** 2023-02-03

**Authors:** Getiye Dejenu Kibret, Daniel Demant, Andrew Hayen

**Affiliations:** 1 Department of Public Health, College of Health Sciences, Debre Markos University, Debre Markos, Ethiopia; 2 School of Public Health, Faculty of Health, University of Technology Sydney, Ultimo, NSW Australia; 3 School of Public Health and Social Work, Faculty of Health, Queensland University of Technology, Brisbane, QLD Australia

**Keywords:** Distance, Maternal health service, Neonatal mortality

## Abstract

**Introduction:**

In Ethiopia, more than half of newborn babies do not have access to Emergency Obstetric and Neonatal Care (EmONC) services. Understanding the effect of distance to health facilities on service use and neonatal survival is crucial to recommend policymakers and improving resource distribution. We aimed to investigate the effect of distance to health services on maternal service use and neonatal mortality.

**Methods:**

We implemented a data integration method based on geographic coordinates. We calculated straight-line (Euclidean) distances from the Ethiopian 2016 demographic and health survey (EDHS) clusters to the closest health facility. We computed the distance in ESRI ArcGIS Version 10.3 using the geographic coordinates of DHS clusters and health facilities. Generalised Structural Equation Modelling (GSEM) was used to estimate the effect of distance on neonatal mortality.

**Results:**

Poor geographic accessibility to health facilities affects maternal service usage and increases the risk of newborn mortality. For every ten kilometres (km) increase in distance to a health facility, the odds of neonatal mortality increased by 1.33% (95% CI: 1.06% to 1.67%). Distance also negatively affected antenatal care, facility delivery and postnatal counselling service use.

**Conclusions:**

A lack of geographical access to health facilities decreases the likelihood of newborns surviving their first month of life and affects health services use during pregnancy and immediately after birth. The study also showed that antenatal care use was positively associated with facility delivery service use and that both positively influenced postnatal care use, demonstrating the interconnectedness of the components of continuum of care for maternal and neonatal care services. Policymakers can leverage the findings from this study to improve accessibility barriers to health services.

**Supplementary Information:**

The online version contains supplementary material available at 10.1186/s12913-023-09070-x.

## Introduction

The distance to the nearest health facility influences the uptake of maternal and neonatal health services [1]. Lack of access to healthcare facilities may cause delays in timely care and influence maternal service utilisation due to associated travel costs and suffering [2], thereby raising the risk of newborn mortality [3]. Only a few studies have investigated the effects of distance to health facilities on neonatal mortality in low and middle-income countries and revealed the influence of distance on neonatal survival [4, 5]. A study from Zambia and Bangladesh showed that a lack of geographic access to emergency obstetric care services is a key determinant factor for the use of facility-based delivery [5, 6].

The Zambian study indicated that every doubling of distance to the nearest facility resulted in a 29% decrease in facility delivery [5]; in Bangladesh, every five-minute increase in travel time to the closest emergency obstetric care facility decrease the likelihood of facility delivery by 30% [6].

**Fig. 1:**
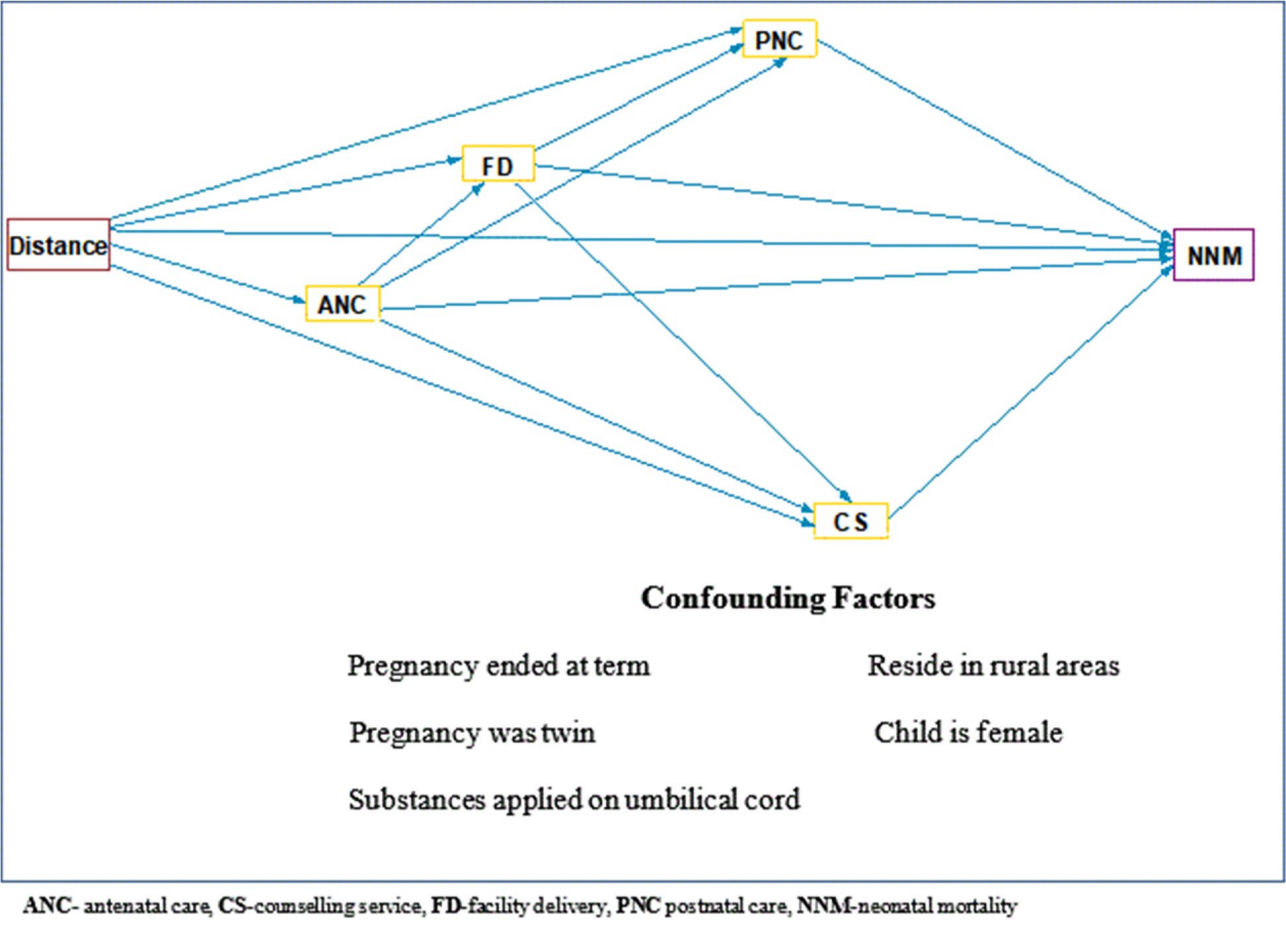
Conceptual framework showing the relationship of distance to health facilities, antenatal care use, skilled delivery, postnatal care and neonatal mortality

Studies have also shown that increased distance between home and health services contributed to neonatal and childhood mortality [7–10]. A systematic review in low and middle-income countries found that distance to health facilities is a significant predictor of under-5 mortality, particularly during the perinatal and neonatal periods [11]. However, other literature reported no association between distance to healthcare facilities and neonatal mortality. There has also been reported elsewhere that neonatal mortality decreased as the distance from home to health facilities increased [4]. However, the researchers cautioned that the result might be affected by the quality of the data. These inconsistent findings might also be attributed to inappropriate modelling of the relationship between distance, maternal service use and neonatal mortality. Some studies considered antenatal care attendance, place of delivery, and postnatal care at the same level with distance [4]. Instead, the relationship between distance to health services and neonatal mortality can be better investigated by taking health service use factors into account as an intermediary between distance and neonatal mortality (see Fig. 1).

The conceptual framework presented in Fig. 1 is based on the components of the continuum of care for maternal and neonatal care integrating health service use during pregnancy, birth and immediately after birth, with the success of each service building upon the previous stage's success. Antenatal care attendance to a skilled health care provider can avert problems during pregnancy and increase the possibility of attending a facility delivery [12]. Giving birth at a health facility again promotes postnatal health service visiting and postnatal counselling about neonatal danger signs [13]. Inadequate service use in the continuum breaks the critical link in the continuity of care and can affect women's and their children's health outcomes [12].

In low-income nations such as Ethiopia, where most of the population lives in rural areas with limited infrastructure, the distance to health care is out of reach for many households. Despite the positive efforts made by the Ethiopian government to improve health outcomes by boosting the number of health workers and expanding health facilities, health service utilisation remains low. In 2016, only 31.8% of Ethiopian pregnant women received prenatal care, with 26.2% of deliveries taking place in health facilities [14].

Ethiopia has had persistently high neonatal mortality rates with considerable disparities by geography and administrative regions [14, 15]. In our previous study, we found that people experienced difficulties accessing emergency newborn care, with only 46.5% of all births in Ethiopia having access to emergency neonatal care facilities within two hours of travel time. The geographical distribution of healthcare facilities as, well as availability of roads, transport and communication are also highly variable across administrative regions and geographic areas. Addis Ababa and Harari administrative regional states had 100% emergency newborn care services coverage, while the Afar and Somali regional states had the lowest access with only 15.3% and 16.3%, respectively [16]. This indicates that pregnant women experience long distances to travel and challenging transportation conditions to access emergency neonatal care services.

The current literature on neonatal mortality in Ethiopia showed that determinants can be grouped into sociodemographic, healthcare-related, obstetric, and medical factors. Key contributing factors to neonatal mortality include area of residence, wealth, education, birth interval, maternal age, tetanus toxoid vaccination, breastfeeding, maternal health service use, place of birth and facility type, the performance of EmONC functions, premature rupture of membrane, gestational age, twin pregnancy, baby size, birth complications, mode of delivery, skilled birth attendance and postnatal health services use [17–21]. We have identified key contributing factors to neonatal mortality in prior work on factors contributing to neonatal mortality that included a survey-based comprehensive analysis of socioeconomic, healthcare, and geographic factors [22]. In the current study, we have incorporated all significant contributing factors from the previous analysis. This study aimed to explore the effect of distance to health facilities on maternal service uptakes and neonatal mortality in Ethiopia.

## Methods and materials

### Population and outcome measurement

We included neonates aged ≤ 28 days from women's most recent deliveries in the prior five years from the 2016 Ethiopian EDHS. We included the most recent births since some data on maternal health services use, such as antenatal and postnatal care, were only available for this population. The primary outcome variable was neonatal mortality, and the primary exposure variable was distance to health facilities that provide maternal and newborn health services. We considered antenatal care, facility delivery, postnatal care and postnatal counselling services as mediator variables. Term of pregnancy, application of substances on the umbilical cord, place of residence, gender of the neonate and the type of pregnancy (twin/singleton) were considered as covariates.

### Data source and extraction

The data for this analysis were obtained from two health-related surveys: the Ethiopian 2016 Emergency Obstetric and Neonatal Care (EmONC) survey [23] and the Ethiopian 2016 EDHS [14]. The outcome variable, mediating variables, socio-economic covariates and the corresponding Geographic Positioning System (GPS) coordinates were extracted from the DHS data, while the distance variable and facility location GPS coordinates were extracted from EmONC dataset. The EmONC assessment survey was a national cross-sectional census of health facilities providing maternal and newborn health services. A total of 3,804 geo-referenced health facilities were included in the survey. A detailed description of the EmONC survey procedure is available in the main report [23]. The EDHS is a cross-sectional survey of nationally representative samples. In the DHS surveys, samples were selected using a stratified, two-stage cluster design, using enumeration areas (clusters) as primary sampling unit and households as secondary sampling unit. The detailed methodology is found in the final EDHS 2016 report [14].

### Data analysis

Based on the DHS recommendations [24], sample weighting was applied to compute frequencies and percentages of neonatal mortality and maternal health service use variables. The analysis was carried out in four steps.

- 1) We joined data from the DHS and EmONC surveys using geographic coordinates recorded in the datasets. The EmONC survey was a census of all health facilities providing maternity and newborn health services (private clinics, health centres and hospitals). For the DHS, the GIS program automatically excluded 21 clusters that lacked spatial references, resulting in 622 clusters with a total of 6941 live births joined with 480 health facility coordinates. The households in the same cluster were assumed to share the same access level to the closest health facility.
- 2) Distance from the centre of the DHS cluster to the closest health facility was computed. The Euclidean distance in kilometres was calculated in ESRI ArcGIS Version 10.3 [25] using the geographic coordinates of DHS clusters and EmONC health facilities. Euclidean distance is a line segment between two points in coordinate geometry [26]. The length of a segment connecting the two points is calculated as:
  $$d =\sqrt{{\left({x}_{1}-{x}_{2}\right)}^{2}+{\left({y}_{1}-{y}_{2}\right)}^{2}}$$

where,

$({x}_{1},{ y}_{1})$ are the coordinates of one point (e.g., the centre of the cluster)

$({x}_{2},{ y}_{2})$ are the coordinates of the other point (the location of the health facility) and

$d$ is the distance between $({x}_{1},{ y}_{1})$ and $({x}_{2},{ y}_{2})$

- 3) The Box-Tidwell test was performed to check the linearity between the distance variable and the logit of neonatal death (see S1). The Box-Tidwell test is used to check for linearity between the continuous predictors and logit of the outcome variable. This is done by adding log-transformed interaction terms between the continuous independent variables and their corresponding natural logarithm into the model. If these interaction terms are significant, there is nonlinearity in the logit [27]. In our data, the linearity assumption between the distance variable and the logits of the outcome variables held for each model.
- 4) We constructed the theoretical model in terms of a diagram constructing the path diagram based on our hypothesis about the relationship between variables. As presented in the conceptual framework (Fig. 1), the effect of distance on neonatal mortality was modelled considering antenatal care, facility delivery and postnatal care use as mediating variables and socio-economic variables as potentially confounding variables. A mediator is a variable in a causal sequence between two variables and transfers the effect of an explanatory variable on the outcome variable [28]. In order to carry out this analysis, the Generalised Structural Equation Modelling (GSEM) method was used. Structural equation modelling (SEM) is a versatile multivariate technique that uses a conceptual model, a path diagram, and a system of connected regression-style equations to represent complex and dynamic interactions in a web of variables [29]. GSEM is a family of SEM statistical techniques that accommodates a discrete outcome variable, which is the case in this study (death vs survival).

In order to check clustering effects, we fit and compared multiple models: 1) a model without clustering effect, 2) considering administrative region as a clustering effect, 3) considering enumeration area as a clustering effect, 4) considering nesting of clusters in regions as a clustering effect and we used the fourth step as a final model. We have checked for collinearity between variables using variance inflation factor, and no collinearity was detected.

## Results

We included a total of 6,941 (51.8% male and 48.2% female) most recent live births in the five years before the 2016 EDHS survey. There were 142 neonatal deaths in the study population, with a mortality rate of 21.5 (95% CI: 18.4, 25.1) per 1,000 live births. The neonatal mortality rates within the first, second, third and fourth quartiles of distance were 16.4 (95% CI:14.1 to 23.6), 18.2 (95% CI:12.8 to 25.7), 20.7 (95% CI:15.0 to 28.6), and 26.5 (95% CI: 19.9 to 35.2) per 1000 live births, respectively. Only 31.7% of births took place in health facilities, and 1057 (14.2%) of mothers applied dung, oil, butter or ointment items on their child's umbilical cord during the days following birth (see Table 1). The Euclidean distance in kilometres was computed between the 622 clusters and the nearest 480 health facilities. The median distance from the centre of clusters to health facilities was 3.8 km (Interquartile range: 1.5 km to 6.9 km). The shortest distance from the centre of a cluster to a health facility was 50 m, and the longest distance was 49 km. All the live births that were residing in areas farther than 18 km from health facilities were from emerging regional states (Somali, Afar, Benishangul Gumuz and Gambella regional states) and no live births of the other regional states reside further than 18 km.

**Table 1:** Maternal Health service use and other characteristics of the study population, Ethiopian demographic and health survey, 2016

Variable	Category	Weighted Frequency	Proportion	95% Confidence Intervals
Antenatal care attendance (4 +)	No	5,041	67.9	66.3, 69.5
	Yes	2,381	32.1	30.5, 33.7
Provide delivery at Health facility	No	5,068	68.3	66.7, 69.9
	Yes	2,354	31.7	30.1, 33.3
Postnatal care within the first 2 days	No	6,299	84.9	83.7, 86.0
	Yes	1,123	15.1	14.0, 16.3
Got counselling about neonatal danger signs within 2 days of birth	No		12.7	11.9, 13.5
	Yes	884	87.3	86.5, 88.0
Gestational age	Term	7323	98.7	98.3, 98.9
	Preterm	99	1.3	1.1, 1.6
Sex of child	Male	3846	51.8	50.7, 52.9
	Female	3576	48.2	47.0, 49.3
Place of residence	Urban	964	13.0	12.2, 13.8
	Rural	6458	87.0	86.2, 87.8
Type of pregnancy	Singleton	7305	98.4	98.1, 99.7
	Twin	117	1.6	1.3, 1.9

### Effect of distance on maternal service use and neonatal mortality

Distance to health facilities was associated with the rate of neonatal mortality and maternal health services utilisation during pregnancy, birth and immediately after birth. For every ten km increase in distance to a health facility, the odds of neonatal mortality increased by 1.33 (95% CI: 1.06 to 1.67). The odds of neonatal mortality increased by 6%, 15% and 22% for every two km, five km and seven km distance from a nearby health facility. Distance is also associated with antenatal care, facility delivery, and postnatal counselling service use. For every ten km increase in distance, the odds of facility delivery decreased by 8.0% (95% CI: 6.0% to 10.0%). For a ten km increase in distance from a health facility, the odds of antenatal care use decreased by 10% (95% CI: 8.0% to 12.0%) [see S2a, b, showing the neonatal mortality and health services use as a function of distance]. Factors such as term pregnancy (AOR = 0.09, 95% CI: 0.05 to 0.16), child with female gender (AOR = 0.55, 95% CI: 0.38 to 0.78), residing in rural areas (AOR = 2.41, 95% CI: 1.31 to 4.42) and twin pregnancy (AOR = 3.04, 95% CI: 2.28 to 4.04) had significantly influenced the rate of neonatal mortality (see Table 2).

**Table 2:** Effect of distance on maternal health services use and neonatal mortality (using generalised structural equation modelling) in Ethiopia, EDHS 2016

**Explanatory variable**	**Outcomes variable**	Adjusted Odds ratio	**95% CI**	***P*-value**
Distance to a health facility (per 10 km)	Antenatal care	0.90	(0.88, 0.92)	< 0.01
Residing in rural areas		0.72	(0.70, 0.74)	< 0.01
Twin pregnancy		1.05	(1.01, 1.09)	0.02
Distance to a health facility (per 10 km)	Facility delivery	0.92	(0.90, 0.94)	< 0.01
Antenatal care		1.33	(1.31, 1.36)	< 0.01
Term pregnancy		0.98	(0.91, 1.05)	0.58
Residing in rural areas		0.68	(0.66, 0.69)	< 0.01
Twin pregnancy		1.00	(0.96, 1.04)	0.99
Distance to a health facility (per 10 km)	Postnatal care	0.98	(0.98, 1.01)	0.83
Antenatal care		1.06	(1.04, 1.08	< 0.01
Facility delivery		1.51	(1.48, 1.54)	< 0.01
Term pregnancy		1.01	(0.99, 1.00)	
Female gender of child		1.01	(0.99, 1.02)	0.73
Residing in rural areas		0.94	(0.92, 0.96)	< 0.01
Twin pregnancy		1.01	(0.99, 1.05)	0.27
Distance to a health facility (per 10 km)	Counselling service	0.97	(0.94, 0.99)	0.018
Antenatal care		1.07	(1.03, 1.11)	< 0.01
Facility delivery		1.14	(1.09, 1.18)	< 0.01
Term pregnancy		1.03	(0.91, 1.16)	0.69
Female gender of child		0.98	(0.95, 1.01)	0.24
Residing in rural areas		0.88	(0.84, 0.92)	< 0.01
Twin pregnancy		1.05	(0.98, 1.11)	0.17
Distance to a health facility (per 10 km)	Neonatal mortality	1.33	(1.06, 1.67)	0.01
Antenatal care		0.78	(0.51, 1.18)	0.24
Facility delivery		1.16	(0.72, 1.89)	0.54
Postnatal care		1.10	(1.64, 1.90)	0.72
Counselling about neonatal danger signs		0.97		0.81
Term pregnancy		0.09	(0.05, 0.16)	< 0.01
Substances applied on umbilical cord		1.07	(0.99, 1.17)	0.09
Female gender of child		0.55	(0.38, 0.78)	< 0.01
Residing in rural areas		2.41	(1.31, 4.42)	< 0.01
Twin pregnancy		3.04	(2.28, 4.04)	< 0.01

Reference group for variables in the table: Attend postnatal care = not attended, counselling = didn't get counselling, facility delivery = didn't deliver at a health facility, attend ANC = not attended 4 or more, Term pregnancy = preterm, Substances applied on umbilical cord = nothing applied, child sex = female, residence = urban, pregnancy was twin = singleton

Attending four or more antenatal care sessions (AOR = 1.06, 95% CI: 1.04 to 1.08) and giving delivery at a health facility (AOR = 1.51, 95% CI: 1.48 to 1.54) were found to influence postnatal care service use. Those who attended four or more antenatal care were more likely to receive counselling services about newborn danger signs (AOR = 1.07, 95% CI: (1.03 to 1.11) and were 33% (95% CI: 31.0% to 36.0%) more likely to give facility delivery than their counterparts after controlling for other confounding factors. The odds of receiving counselling services was higher among those who attended facility delivery than those who did not give birth at a health facility (AOR = 1.14, 95% CI: 1.03 to 1.11).

## Discussion

The distance between residence and health-care facilities was found to have an impact on neonatal mortality. However, while use of health services and application of substances to umbilical cords were determinants of neonatal death in the prior study, they are not statistically significant in our current analysis. Closer proximity to health facilities was associated with lower neonatal mortality. These finding is in agreement with a previous study in Ethiopia [30] that found closer proximity to health facilities was associated with lower early neonatal mortality, as well as studies from Vietnam [8] and India [31] that showed closer proximity to health facilities had an association with lower neonatal mortality. However, our finding is inconsistent with a study conducted in Malawi and Zambia that showed no association between distance and neonatal mortality in Malawi, while in Zambia, ten km further distance was associated with lower neonatal mortality [4]. A finding from India also showed that neonatal mortality decreased as the distance from home to primary health care services increased [31]. This disparity could be related to a difference in statistical approach, as we examined the mediated effect of distance on neonatal mortality, whereas the former studies considered distance variables at the same level with maternal health service use variables. The other explanation could be that the difference in the quality of services among different countries.

There was no significant association between neonatal mortality and maternal health services use (ANC, FD and PNC). However, previous studies showed that antenatal care use and the place of delivery influenced neonatal mortality [8].

We also investigated distance to health facilities and its influence on the utilisation of antenatal care, facility delivery and postnatal care services. Our findings showed that the farther the distance to health services, the lower the utilisation of antenatal, perinatal and postnatal counselling services. In agreement with our findings, studies from Kenya [32, 33], Ghana, Malawi and Zambia showed that further distance to care was negatively associated with facility use for delivery services [4, 34–37]. Distance to health facilities was also associated with lower use of antenatal care or a delay in attending ANC visits. In support of our finding, a systematic review in sub-Saharan Africa showed that further distance to health facilities reduces the uptake of antenatal care service use [38]. Another study from northwest Ethiopia showed that distance to health facilities caused a delay in ANC attendance [39], and a study in Burkinafaso found that proximity increased the odds of getting four or more ANC visits[36]. This is because distance can determine the accessibility to health facilities, cause delays to care, and influence the ability to use health services during pregnancy, at birth, and during the postnatal period [2].

Attending four or more antenatal care was found to influence facility delivery, getting counselling services and postnatal care service use. A systematic review in Ethiopia found that mothers who received one or more antenatal care visits were more likely to deliver at health facilities and use postnatal care services, which was consistent with our findings [13]. Studies from the Ethiopian DHS on factors associated with facility delivery and postnatal care service use also showed that attending four or more ANC was found to increase the probability of facility delivery and postnatal care use [40, 41]. This is because ANC is an important entry point for subsequent use of delivery and PNC services; hence, it promotes women to have a birth preparedness and complication readiness plan [42] and then give birth at a health facility and return for a postnatal visit [13, 43].

We found that women residing in rural areas were less likely to use antenatal care, facility delivery, and counselling services on neonatal danger signs and postnatal care services than women residing in urban areas. In agreement with our finding, a study from the 2011 Ethiopian DHS further analysis showed that women from urban areas were more likely to use ANC, facility delivery and postnatal care services than women from rural areas [41]. A systematic review and meta-analysis in low- and middle-income countries also showed that postnatal care service use was lower among women from rural areas [44]. This can be related to the health services accessibility and better infrastructure coverage in urban areas. The rural areas in Ethiopia suffer from shortages of basic health services, road, electricity and water infrastructures [45]. We found that giving delivery at a health facility increased the probability of postnatal care service use. A study from Uganda on determinants of early postnatal care use showed that facility delivery was associated with increased uptake of postnatal care service use [46]. It can be because health professionals can educate women and their families about healthy parenthood and the necessity of postnatal care during birth. Even though it was not statistically significant, the association between preterm pregnancy and maternal health service use was strange. Preterm births were less likely to seek postnatal care and counselling services than term births. This could be related to mothers' fear of social isolation due to having small babies and unfavourable attitudes about their baby in the early days of birth [47].

### Implications

We implemented a data integration method based on geographic coordinates that can be applied to other settings and contexts. Because of the growing availability of geo-referenced health and related data, this approach would give an excellent opportunity to support evidence-based policy initiatives.

The findings have implications for health care planning in Ethiopia and comparable settings in low-income countries. We found out that long-distance impacted health services use during pregnancy and immediately after birth which signals the need to design a strategy to improve access to basic maternal and neonatal health services. In this regard, increasing the health force work and the number of health facilities in rural areas can help. The health extension program in Ethiopia has shown tangible positive impacts on community health improvements. Upgrading the health posts to health centres with basic maternal and neonatal health services and adequate health extension workers' training would significantly improve the accessibility problem.

Evidence also suggested innovative strategies would help to enhance health services access, and utilisation as well as close the geographic disparities in health services coverage in LMICs. Priorities included strategies for health service delivery, medical products and health technologies, health workforce, health care financing, community ownership and participation, and leadership and governance [48]. In this regard, strengthening some of the strategies that have been implemented in Ethiopia to improve maternal and neonatal health, such as community-based treatment of sick newborns, emergency transportation, and women’s developmental army (women’s groups and community-based intervention packages), would be helpful [49].

In addition, introducing strategies such as providing vouchers for maternal health service-related costs, including transportation, should be considered as well as other strategies to improve access through obstetric insurance schemes, community-based emergency loans, and positive deviance approaches to improve antenatal nutrition and changing health-seeking behaviour [48].

### Strength and limitations of the study

We implemented a data integration method from two nationally representative surveys based on geographic coordinates. The study examined the effect of distance on neonatal mortality, controlling for confounders identified from previous related studies on determinants of neonatal mortality. However, our study has some limitations to consider when interpreting the results. All households in a cluster were assumed to have equal access levels to the nearest health facility, and the geographical displacement of DHS survey locations for privacy reasons may have an impact on interpretation. There is a possibility that maternal care could be obtained outside the nearest health facility, which will overestimate the relationship between distance to the nearest health facility and maternal service use. The use of straight (Euclidean) distance measurement could not also take landscape barriers into account. We did not also analyse the effect of quality of services on healthcare utilisation and neonatal mortality in addition to the effect of distance.

## Conclusions

Distance to health services was found to impact the use of maternal services (such as antenatal care visits, perinatal care service use) as well as neonatal survival. A longer distance to the nearest health facility can be an obstacle to seeking care. In Ethiopia, majority of the community are disadvantaged as they often lack road infrastructure and transportation. Antenatal care use was associated with facility delivery service use, whereas both antenatal care and facility delivery services positively influenced postnatal care. The GSEM model simultaneously depicts the relationship between distance, maternal health service utilisation and neonatal mortality. The association between distance and infant mortality was shown to be mediated by factors such as antenatal care use, facility delivery, and postnatal counselling services. This supports our hypothesis that maternal health service use mediates the relationship between distance and neonatal mortality. Policymakers can leverage the findings from this study to improve health services coverage. Increased access to maternity and neonatal health care should be prioritised in the rural community. Policymakers and programs should contemplate mapping the updated distribution of health care facilities, road and emergency transport access, and population groups beyond the timely reach of emergency newborn care. Further consideration should be given to geographic disparities when planning facility expansions and service distribution. Finally, it would be beneficial to investigate the impact of quality on maternal service utilisation in addition to geographic access.

## Supplementary Information

**Additional file 1:** **S1.** Box-Tidwell test to check for linearity between distance and log transform of neonatal death.

**Additional file 2:** **S2** a. Facility-based delivery as a function of distance to health facilities. b Neonatal mortality as a function of distance to health facilities.

## Data Availability

The data that support the findings of this study are available from the DHS Program database accessed from https://dhsprogram.com/Data/ and from The Ethiopian Public Health Institute, where restrictions apply to the availability of these data, which were used under license for the current study, and so are not publicly available. Data are, however, available from the authors upon reasonable request and with permission of the Ethiopian Public Health Institute.

## Abbreviations

AOR: Adjusted Odds Ratio
DHS: Demographic and Health Survey
EmONC: Emergency Obstetric and Neonatal Care
GIS: Geographic Information System
GSEM: Generalised Structural Equation Modelling
KM: Kilometre
