# Supplementary material for: The effect of distance to health facility on neonatal mortality in Ethiopia
Source: BMC Health Serv Res. 2023 Feb 3;23:114. doi: 10.1186/s12913-023-09070-x (PMC9896723; doi:10.1186/s12913-023-09070-x)
Supplement: Supplementary file 1 — Additional file 1: S1. Box-Tidwell test to check for linearity between distance and log transform of neonatal death. [file 12913_2023_9070_MOESM1_ESM.docx]

**S1: Box-Tidwell test to check for linearity between distance and log transform of neonatal death**

The null hypothesis for the Box-Tidwell test is that the predictor variable **distance** is of a linear term, or, equivalently, p1 = 1. As p1 goes far from 1, the figure suggests the possible transformation method to improve the model fitness. Fortunately, in our model, the Box-Tidwell test of nonlinearity for the variable **distance**is not statistically significant (p-value = 0.843), and p1 is close to 1, which indicates the null hypothesis holds there is a linear relationship between the distance variable and log transformation of the outcome variable (neonatal death).
