# Supplementary material for: The effect of distance to health facility on neonatal mortality in Ethiopia
Source: BMC Health Serv Res. 2023 Feb 3;23:114. doi: 10.1186/s12913-023-09070-x (PMC9896723; doi:10.1186/s12913-023-09070-x)
Supplement: Supplementary file 2 — Additional file 2: S2 a. Facility-based delivery as a function of distance to health facilities. b Neonatal mortality as a function of distance to health facilities. [file 12913_2023_9070_MOESM2_ESM.docx]

The figures in the supplementary file (S2a and S2b) show the trends of facility delivery and neonatal mortality as a function of distance in Kilometres (KMs). The facility-based delivery declines as distance increases and neonatal mortality increases as distance increases.

**S2a: Facility-based delivery as a function of distance to health facilities**

**S2b: Neonatal mortality as a function of distance to health facilities**
